# Colony Stimulating Factors in Early Feline Infectious Peritonitis Virus Infection of Monocytes and in End Stage Feline Infectious Peritonitis; A Combined In Vivo and In Vitro Approach

**DOI:** 10.3390/pathogens9110893

**Published:** 2020-10-27

**Authors:** Alexandra J. Malbon, Eleni Michalopoulou, Marina L. Meli, Emi N. Barker, Séverine Tasker, Keith Baptiste, Anja Kipar

**Affiliations:** 1 Institute of Veterinary Pathology, Vetsuisse Faculty, University of Zurich, 8057 Zurich, Switzerland; anja.kipar@uzh.ch; 2 Center for Clinical Studies, Vetsuisse Faculty, University of Zurich, 8057 Zurich, Switzerland; mmeli@vetclinics.uzh.ch; 3 Department of Livestock and One Health, Institute of Infection, Veterinary and Ecological Sciences, University of Liverpool, Neston CH64 7TE, UK; e.michalopoulou@liverpool.ac.uk; 4 Clinical Laboratory, Department of Clinical Diagnostics, and Services, Vetsuisse Faculty, University of Zurich, 8057 Zurich, Switzerland; 5 Bristol Veterinary School, University of Bristol, Langford, Bristol BS40 5DU, UK; emi.barker@bristol.ac.uk (E.N.B.); s.tasker@bristol.ac.uk (S.T.); 6 Langford Vets, University of Bristol, Langford, Bristol BS40 5DU, UK; 7 The Linnaeus Group, Shirley, Birmingham B90 1BN, UK; 8 Department of Veterinary Medicine, Danish Medicines Agency (Lægemiddelstryelsen), Axel Heides gade 1, DK-2300 Copenhagen South, Denmark; keb@dkma.dk; 9 Department of Infection Biology and Microbiomes, Institute of Infection, Veterinary and Ecological Sciences, University of Liverpool, Liverpool L3 5RF, UK

**Keywords:** feline coronavirus, feline infectious peritonitis, monocyte proliferation, colony stimulating factors

## Abstract

Feline coronavirus (FCoV) infection initiates monocyte-associated viremia and viral persistence. Virus-infected, -activated monocytes also trigger feline infectious peritonitis (FIP), a fatal systemic disease of felids typified by granulomatous (peri)phlebitis. Currently, the exact mechanisms inducing monocyte activation and FIP are unknown. This study attempted to identify the potential immediate effect of virulent FCoV on colony-stimulating factor (CSF) (granulocyte (G)-CSF, monocyte (M)-CSF and granulocyte-monocyte (GM)-CSF levels through in vitro assessment, alongside prototypical pro- and anti-inflammatory mediators (interleukin (IL)-1, IL-6, IL-12p40, tumor necrosis factor (TNF)-α, and IL-10); this was assessed alongside the in vivo situation in the hemolymphatic tissues of cats euthanized with natural end-stage FIP. For the in vitro work, isolated monocytes from SPF cats were cultured short-term and infected with the FIP virus (FIPV) strain DF2. Mediator transcription was assessed by quantitative reverse transcriptase PCR (RT-qPCR) at 3, 6 and 9 h post infection (hpi), and in the post-mortem samples of bone marrow, spleen, and mesenteric lymph nodes (MLN) of cats with FIP. We observed limited and transient changes in cytokine transcription in monocytes after infection, i.e., a significant increase of IL-6 at 3 hpi and of GM-CSF over the 3 and 6 hpi period, whereas M-CSF was significantly decreased at 9 hpi, with a limited effect of age. The findings indicate that the infection induces expansion of the monocyte/macrophage population, which would ensure the sufficient supply of cells for consistent viral replication. In natural disease, the only upregulation was of G-CSF in the MLN, suggesting either immune exhaustion or an active downregulation by the host as part of its viral response.

## 1. Introduction

Feline infectious peritonitis (FIP) is a widely distributed feline coronavirus (FCoV)-induced systemic disease in cats, characterized by a granulomatous to necrotizing phlebitis and periphlebitis, granulomatous inflammatory lesions in several organs, and fibrinous to granulomatous serositis with protein-rich body cavity effusions [1,2,3]. Following viral transmission via the fecal–oral route and enterocyte infection [4], most infected cats develop a monocyte-associated FCoV viremia with some degree of viral replication in monocytes [5,6,7,8]. Monocytes are the cells that are responsible for viral dissemination and the development of FCoV persistence in tissues [9]. When activated, infected monocytes mediate granulomatous phlebitis and periphlebitis, the disease process causing the typical FIP lesions [1], with accumulation of abundant macrophages at lesion sites. Alongside the other features, both FCoV-infected cats without FIP and cats with FIP exhibit evidence of the proliferation and activation of monocytes/macrophages and their precursors in hemolymphatic tissues [10,11]. Inflammatory cytokines and chemokines likely target these cells, leading to their activation and to lesion development; a separate process by which sufficient cells are produced in the bone marrow or at extramedullary sites, which can also reasonably be presumed to occur in parallel to ensure a sufficient supply of monocytes/macrophages for viral replication and lesion development.

Feline monocytes constitutively transcribe cytokines, such as interleukin (IL)-1β, IL-6, IL-10, IL-12p40 and tumor necrosis factor (TNF)-α. Levels of transcription are variable but generally highest for IL-1β [12]. They also change with age, when a general drop in constitutive transcription is seen in middle age, compared to young and older age [11]. In association with the development of FIP phlebitis, monocytes show strong TNF-α expression alongside upregulation of adhesion molecules [1]; FIPV-infected monocytes were also found to express vascular endothelial growth factor (VEGF) [13].

The colony stimulating factors (CSFs) are members of the cytokine superfamily, first named numerically for their ability to act as growth factors for hematopoietic cells in vitro. They have since been renamed monocyte (M)-CSF, granulocyte monocyte (GM)-CSF and granulocyte (G)-CSF, respectively. The CSFs have overlapping but non-redundant roles. M-CSF is important for homeostasis, being detectable in plasma and constitutively expressed by many cell types, including macrophages, endothelial cells, and fibroblasts [14]. GM-CSF is more involved with inflammation and as such is mainly produced by activated leukocytes, including monocytes and macrophages [15]. A slightly oversimplified version of the effect of M-CSF and GM-CSF on mature cells is that they induce an anti- or pro-inflammatory state respectively in macrophages [15], which is also reflected by the fact that M-CSF has a role in increasing cell numbers without altering their activation status [14]. G-CSF has a pleiotropic role which appears to vary from pro- to anti-inflammatory in different situations and models [16]. Its main sources are monocytes and macrophages, as well as other cells including fibroblasts, endothelial cells, and bone marrow stromal cells [17]. Its homeostatic levels are hard to detect, but under stress, e.g., infection, levels rise rapidly [15]. By stimulating the mobilization of hematopoietic cells (particularly neutrophils) from the marrow, it is possible that in parallel to providing more inflammatory potential, G-CSF alters the circulating leukocyte pool to a less mature and hence less inflammatory phenotype [16].

Studies using isolated feline alveolar macrophages and/or monocytes have shown increases in G-CSF, GM-CSF, TNF-α and VEGF production at 2–3 days post infection with FIPV [13,18,19]. However, there is a paucity of information as to the immediate effect of FCoV infection on the function of monocytes, which could consequently occur even in low-level viremia. The present study aimed firstly to assess whether FIPV can directly activate feline monocytes. For this purpose, peripheral blood monocytes were isolated from barrier-maintained, FCoV-free cats of variable age, short-term cultured, infected with the FIPV strain DF2 and examined for transcription of IL-1β, IL-6, IL-10, IL-12p40, TNF-α, G-CSF, M-CSF and GM-CSF by quantitative reverse transcriptase PCR (RT-qPCR) in comparison to uninfected monocytes. Secondly, the present study aimed to assess CSF transcription in clinical cases of FIP, by means of post-mortem evaluation of hemolymphatic organs. The bone marrow (BM), spleen and mesenteric lymph node (MLN) were chosen due to the different roles of these organs in the immune system. Since lesions of FIP are not found in the BM, the viral load there most closely reflects viremia [3]. The spleen is directly embedded into the circulation and contains abundant tissue macrophages, and therefore reflects both the monocyte-associated viremia and local macrophage activation; in addition, the spleen occasionally exhibits parenchymal lesions as well. Finally, the MLN is one of the organs which most commonly exhibits FIP lesions, as well as being at the gateway between enteric and systemic infection [20]. The CSF pattern varied across organs, the only increase in FIP being for G-CSF in the MLN. As the organ with the highest viral load, this suggests a direct or indirect viral induction of the cytokine fueling the inflammatory response, or as part of the overexuberant immune response. In contrast, G-CSF was decreased in the BM in FIP, indicating both a lack of systemic stimulation and a possible local attempt to dampen the inflammatory response. M-CSF, the most macrophage-specific CSF, was lower in the BM and spleen, the organs with lower viral loads. This may suggest either that these organs were able to suppress the response or were exhibiting immune exhaustion. 

## 2. Results

### 2.1. Cytokines Including CSFs Are Constitutively Transcribed in Feline Monocytes with High Variability, and Overall FIPV Infection Has a Selective Effect on Their Transcription Levels

**Figure 1:**
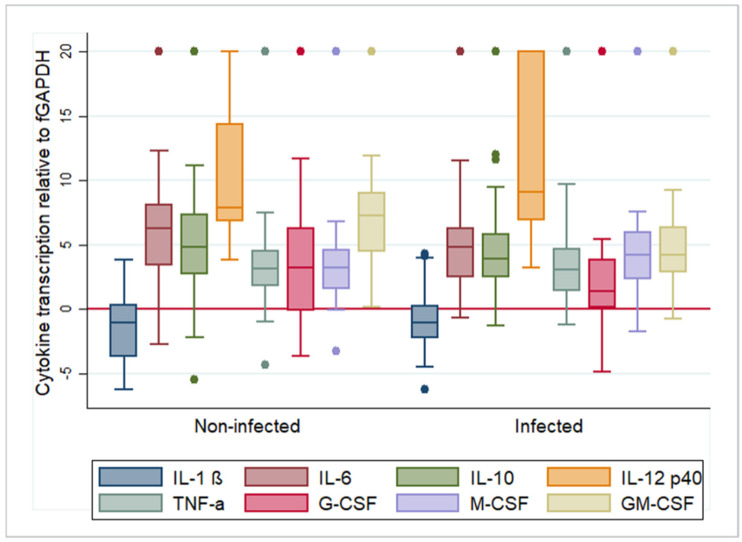
Box and whisker plots illustrating the variability and average relative transcription levels for all examined cytokines, post feline infectious peritonitis virus infection (“infected”) and mock infection (“non-infected”). For each cytokine, results from all animals and time points were pooled. The values are based on the difference in RT-qPCR threshold cycle (ΔCt) values for each cytokine in relation to the house-keeping gene feline (f)GAPDH. Negative values indicate that the cytokine is transcribed at a higher level than GAPDH. The boxes depict the median and interquartile (IQ) range with whiskers extending to the highest and lowest values which are within 1.5× the IQ range. Outliers beyond this are individually marked. Values at 20 ΔCt represent samples where cytokine transcription was not detected. IL-1β/6/10/12p40—interleukin 1 beta/6/10/12p40; TNF-α—tumor necrosis factor alpha; G/M/GM-CSF—granulocyte/monocyte/granulocyte monocyte colony stimulating factor.

Previous studies have shown that constitutive cytokine transcription in isolated feline monocytes (IL-1β, IL-6, TNF-α and IL-12p40) is generally highly variable [12]. The present study examined monocytes that were isolated and cultured using the same approach [12] and then extended the culture after infection with DF2, a virulent FIPV serotype II isolate that induces FIP under experimental conditions [21], or mock infection. The incubation of isolated feline monocytes with DF2 for 1 h, at 15–18 h after plating, led to their infection, as confirmed by RT-qPCR performed at 3, 6 and 9 h post incubation (hpi; data not shown; [22]). We then assessed the inflammatory cytokine levels as markers for activation levels of monocytes and could confirm the highly variable transcription that we had previously reported; we also observed constitutive transcription with a similar extent of variation for the CSFs (Figure 1).

Overall, IL-1β was detected in all samples, TNF-α in 95.2% (118/124 samples), IL-6, M-CSF and GM-CSF in 90.3% (112/124 samples), IL-10 in 88.7% (110/124 samples), G-CSF in 87.9% (109/124 samples) and IL-12p40 in 74.2% (92/124 samples). 

In uninfected monocytes, IL-1β was transcribed at the highest relative levels, followed by TNF-α, G-CSF, M-CSF, IL-10, IL-6, GM-CSF and IL-12p40 (Figure 1). The time of culture after mock infection had a very limited effect. Analysis of variance identified a significant association with time from infection for M-CSF (R^2^ = 0.175, F = 6.27, *p* < 0.01). When transcription was examined pairwise for each time point using a t-test, for G-CSF, transcription levels were significantly higher at 6 hpi (mean ΔCt = 2.010) than at 3 hpi (mean ΔCt = 5.460) (t = 2.332, df = 4, *p* < 0.05). For M-CSF, the opposite was observed; transcription levels were significantly lower at 6 hpi compared to 3 hpi (3 h mean ΔCt = 2.862, 6 h mean ΔCt = 7.886, t = −3.221, df = 42, *p* < 0.01) and 9 hpi (mean ΔCt = 3.299, t = 2.216, df = 35, *p* < 0.05). Supplementary Table S1 summarizes the results of the analysis. Coefficients of variation are included to indicate the extent of variation in the transcription levels. This reflects that all cytokines except IL-1β were transcribed with high inter-individual and inter-test variations. For IL-1β, mRNA levels appeared very consistent throughout. 

In FIPV-infected monocytes, IL-1β (mean ΔCt: −0.954) was again transcribed at the highest relative levels, followed by TNF-α (mean ΔCt: 3.836), G-CSF (mean ΔCt: 3.89), M-CSF (mean ΔCt: 5.349), IL-6 (mean ΔCt: 5.513), GM-CSF (mean ΔCt: 5.609), IL-10 (mean ΔCt: 6.101) and IL-12p40 (mean ΔCt: 11.646) (Figure 1). We found differences in the transcription levels of some cytokines in association with the time post infection. Analysis of variance identified a significant association with time for IL-6 (R^2^ = 1.09, F = 3.61, *p* < 0.05), G-CSF (R^2^ = 0.115, F = 3.83, *p* < 0.05), M-CSF (R^2^ = 0.159, F = 5.59, *p* < 0.01) and GM-CSF (R^2^ = 0.221, F = 8.36, *p* < 0.001). When transcription was examined for each step, IL-6 monocytes exhibited significantly higher levels at 3 hpi (mean ΔCt = 4.322) than at 9 hpi (mean ΔCt = 8.052, t = −2.45, df = 41, *p* < 0.05). For G-CSF, the transcription levels were significantly higher at 6 hpi than at 3 hpi (3 h mean ΔCt = 4.56, 6 h mean ΔCt = 0.564, t = 2.353, df = 42, *p* < 0.05). For M-CSF, the opposite was observed; transcription levels were significantly higher at 3 hpi (mean ΔCt = 2.859) than at 6 hpi (mean ΔCt = 6.762, t = −2.616, df = 42, *p* < 0.05) and at 9 hpi (mean ΔCt = 7.317, t = −4.431, df = 41, *p* < 0.001). GM-CSF transcription was significantly higher at 6 hpi (mean ΔCt = 3.223) compared to both 3 hpi (mean ΔCt = 4.778, t = 2.1142, df = 42, *p* < 0.05) and 9 hpi (mean ΔCt = 9.284, t = −3.19, df = 35, *p* < 0.01). Supplementary Table S1 summarizes the results of the analysis. The coefficients of variation show that also after FIPV infection all cytokines except IL-1β were transcribed with high inter-individual and inter-test variations.

### 2.2. FIPV Infection of Feline Monocytes Has a Limited Effect on the Transcription of Selected Cytokines during the First 9 h Post Infection

Previous in vitro studies have shown that FIPV infection of isolated alveolar macrophages, peripheral blood mononuclear cells (PBMC) and/or monocytes can induce the production of cytokines, such as TNF-α, IL-1β, G-CSF, GM-CSF and VEGF, after 1–3 days, an effect enhanced by incubation with an anti-S (spike) protein antibody [13,19,23,24]. Monocytes/macrophages in FIP phlebitis were also shown to strongly express TNF-α [1]. However, it is also of relevance whether monocytes exhibit functional changes immediately after infection, since FCoV infection generally leads to monocyte-associated viremia and some degree of viral replication [5,6,7,8,25], with subsequent persistent infection in tissue macrophages [9]. The present study therefore examined cytokine transcription in monocytes in the early phase after infection (3–9 hpi), around the first viral replication cycle [26].

**Figure 2:**
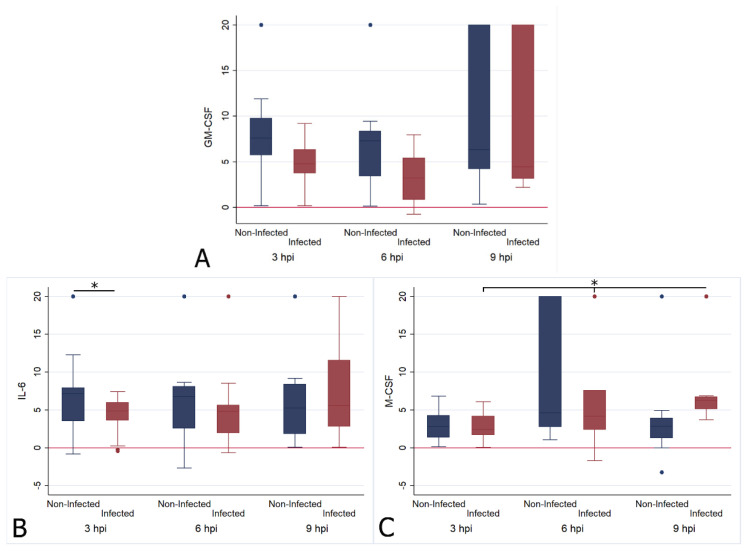
Box and whisker plots illustrating the changes in transcription levels for GM-CSF (**A**), IL-6 (**B**) and M-CSF (**C**) at 3 (n = 25), 6 (n = 19) and 9 (n = 18) hours post FIPV infection (“infected”) in comparison to mock infection (“non-infected”). The values are based on the ΔCt values for each cytokine in relation to the housekeeping gene fGAPDH. Negative values indicate that the cytokine was transcribed at a higher level than fGAPDH. The boxes depict the median and interquartile (IQ) range with whiskers extending to the highest and lowest values which are within 1.5× the IQ range. Outliers beyond this are individually marked. Values at 20 ΔCt represent samples where cytokine transcription was not detected. * indicates a *p* value of at least <0.05, with values for each cytokine given in the text.

FIPV infection had a limited effect on the relative cytokine transcription when investigated across all cats, regardless of the animals’ ages or the individual cat. It appeared greatest for GM-CSF, the levels of which were significantly higher after infection at the 3 h (infected mean ΔCt = 4.778; non-infected mean ΔCt = 8.861, t = 3.335, df = 48, *p* < 0.01) and 6 h (infected mean ΔCt = 3.223; non-infected mean ΔCt = 7.176, t = 2.912, df = 36, *p* < 0.01) time points (Figure 2A), with a significant increase from 3 hpi to 6 hpi, followed by a drop towards 9 hpi, at which point the values were significantly lower than at both previous time points (Figure 2A; Supplementary Table S1).

IL-6 levels were significantly higher in infected monocytes at 3 hpi (infected mean ΔCt = 4.322; non-infected mean ΔCt = 6.944, t = 2.320, df = 48, *p* < 0.05), but had returned to levels comparable to those of uninfected monocytes at the later time points (Figure 2B). M-CSF levels were significantly lower in infected monocytes at 9 hpi (infected mean ΔCt = 7.317; non-infected mean ΔCt = 3.299, t = −2.588, df = 34, *p* < 0.05); they had progressively dropped over time, as shown by the fact that levels were significantly lower at 6 hpi and at 9 hpi compared to 3 hpi (Figure 2C). The IL-1 β, TNF-α, IL-10, IL-12p40, IL-10 and G-CSF transcription levels did not differ significantly from those of uninfected monocytes at any time point.

### 2.3. Cytokine Transcription Levels in Feline Monocytes after FIPV Infection Show Limited Age-Related Variation Which Also Affects the Response to Infection

FIP is a disease mainly observed in young cats, up to two years of age [2]. Interestingly, feline monocytes also show age-related differences in constitutive cytokine transcription, with significantly higher mRNA levels for IL-1β, IL-6 and IL-12p40 in both young and old cats vs. middle aged cats, and a decline in IL-10 transcription with age, though without any statistical significance [27]. This suggests a potential for age to affect the reaction of monocytes to FIPV. Therefore, we performed the present study on monocytes from cats with a wide age range (10 months–7 years 9 months). We examined the association of age and time from infection against the difference in cytokine transcription between infected and uninfected cells (ΔΔCt). This allowed us to control for the effect of ad hoc set values when infected and uninfected cells were examined separately. The results are presented in Supplementary Figure S1. There was relatively high variability within all age groups, with certain trends detectable despite this. As a caveat to the age comparisons, there were low numbers in the older cat group, with only two samples available at 9 hpi.

**Figure 3:**
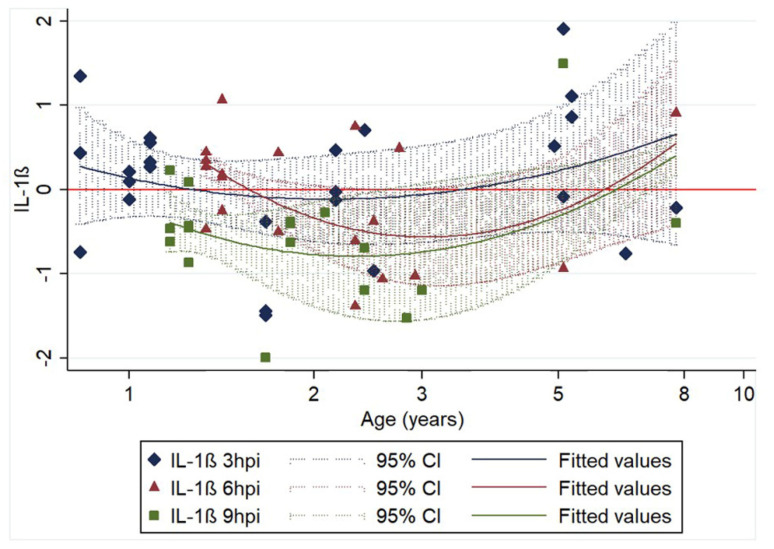
Association of relative IL-1β transcription levels (log10 of ΔΔCt) with age and time of harvesting (3 (25 samples), 6 (19 samples) and 9 (18 samples) hours post infection (hpi)). The 62 data points represent the difference in IL-1β transcription between infected and non-infected cell cultures of the same animal sample. Regression lines (including 95% CIs) are fitted for each time post infection. Regression equation: y = 0.255 − 2.565x + 3.405x^2^ − 0.011z − 0.509ω; n = 62; R^2^ = 0.2027; *p* < 0.05; age (x): *p* < 0.05; age^2^ (x^2^): *p* < 0.01; 6 hpi (z): *p* = 0.956, 9 hpi (ω): *p* < 0.05.

We found that for IL-1β, transcription levels after infection were significantly associated with age. A quadratic model was fitted, and in addition to time from infection, we identified age as a factor associated with relative IL-1β transcription; as observed for constitutive transcription [12], both the young and older cats showed a more intense increase after infection than the middle aged cats. Furthermore, transcription levels were significantly lower at 9 hpi compared to 3 hpi. Figure 3 demonstrates these associations.

**Figure 4:**
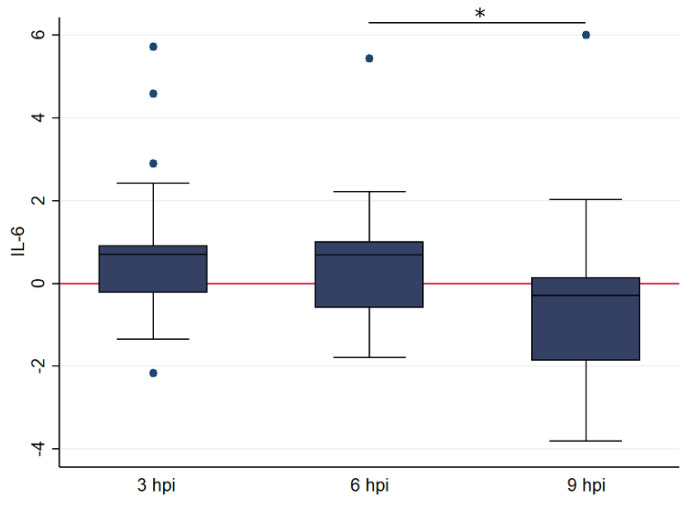
Box and whisker plots illustrating the differences in relative transcription levelsof IL-6 at 3, 6 and 9 h post FIPV infection (with 25, 19, and 18 samples at each time point, respectively). The amount of target was calculated by 2^−ΔΔCT^, using fGAPDH as the internal reference gene for normalization and expressed as an n-fold difference relative to the non-FIP group mean of the BM samples as a calibrator. The boxes depict the median and interquartile (IQ) range with whiskers extending to the highest and lowest values which are within 1.5× the IQ range. Outliers beyond this are individually marked. The boxes depict the median and interquartile (IQ) range with whiskers extending to the highest and lowest values which are within 1.5× the IQ range. Outliers beyond this are individually marked. * indicates a *p* value of at least <0.05, with precise values given in the text.

A similar trend was seen for IL-6, with no significant association with age but with a significantly higher relative IL-6 transcription at 6 hpi (geometric mean: 6.159) compared to 9 hpi (geometric mean: 0.278, t = 2004, df = 41, *p* < 0.05) (Figure 4). No model could be fitted to represent the data.

**Figure 5:**
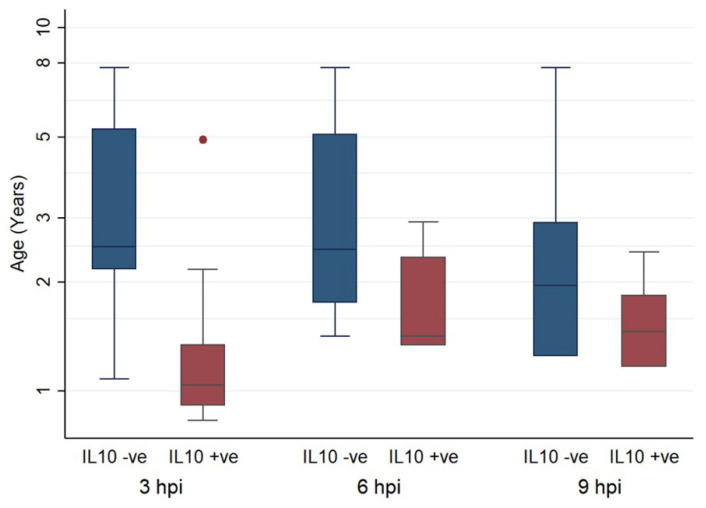
Box and whisker plots comparing the ages of cats in which IL-10 was higher (positive) or lower (negative) in FCoV-infected samples than their paired uninfected samples, at 3, 6, and 9 hpi. The amount of target was calculated by 2^−ΔΔCT^, using fGAPDH as the internal reference gene for normalization and expressed as an n-fold difference relative to the non-FIP group mean of the BM samples as a calibrator. The boxes depict the median and interquartile (IQ) range with whiskers extending to the highest and lowest values which are within 1.5× the IQ range. Outliers beyond this are individually marked.

The age of animals whose monocytes had higher IL-10 transcription post infection than mock infection was significantly lower at 3 hpi and 6 hpi compared to 9 hpi, when examined by univariate analysis (3 hpi: t = 4.054, *p* < 0.001, 6 hpi: t = 4.054, *p* < 0.001, 9 hpi: t = 2.255, *p* = 0.185). This is illustrated in Figure 5. In total, 25 samples were examined at 3 hpi. In 13 (52%), IL-10 was lower in the FCoV-infected monocytes than the uninfected, 19 samples were examined at 6 hpi, from which 6 (31.58%) had lower IL-10 in the infected group, and 18 samples were examined at 9 hpi and in 12 (66.67%) the IL-10 was lower in the infected group.

**Figure 6:**
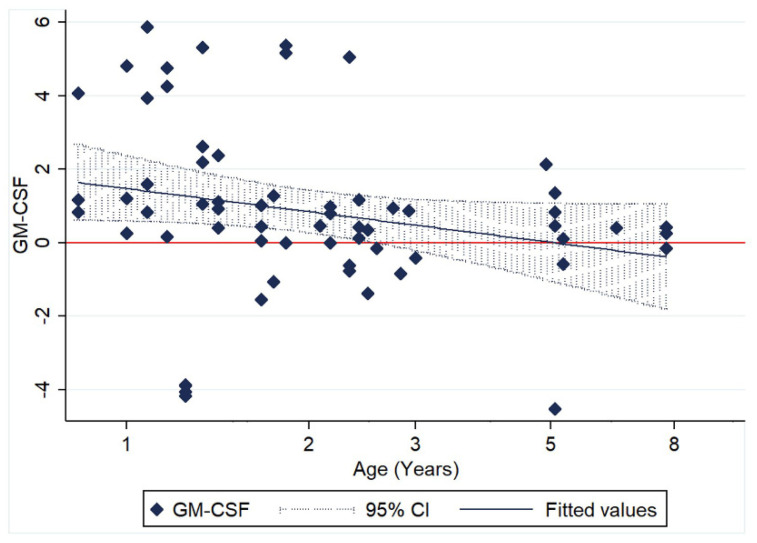
Scatter plot of relative GM-CSF transcription levels (log10 of ΔΔCt) of 62 paired monocyte samples at different time points after FIPV infection (3, 6 and 9 hpi) and in relation to age.

No significant association with age was identified for any of the other cytokines; however, GM-CSF relative transcription demonstrated a trend of declining with age, without any significant association with the time post infection (Figure 6).

The relative transcription (ΔΔCt) of M-CSF showed significantly lower levels at 9 hpi than at both 3 hpi (t = 2.74, df = 41, *p* < 0.01) and 6 hpi (t = 2.876, df = 35, *p* < 0.01). For G-CSF, they were found to be significantly higher at 6 hpi compared to 9 hpi (t = 2.121, df = 35, *p* < 0.05), and for TNF-α they were significantly higher at 3 hpi than at 6 hpi (t = 2.091, df = 42, *p* < 0.05).

### 2.4. Hemolymphatic Organs of Cats with FIP

Our group has previously studied levels of inflammatory cytokines in hemolymphatic organs [11,28], and found that although inflammatory cytokine upregulation could be observed in many FIP cases, particularly in the MLN, the spleen and BM were less involved in the pro-inflammatory response. The present study focusses on the CSFs to further characterize this process since we found that their selective dysregulation is part of the early response of monocytes to FIPV, and that these organs harbor large numbers of macrophages when FIP develops [10]. The presence of virus, and in positive cases the relative viral loads, were evaluated in order to compare infection levels between organs and determine whether this related to CSF levels. In the case of the spleen there was only one pairwise comparison necessary, between the 14 FCoV-positive cats with FIP and the six FCoV-negative non-FIP cats. In the BM, 2 of the 11 cats with FIP were found to be FCoV-negative by RT-qPCR, and an additonal comparison was therefore performed to separate disease and local viral effect. In contrast, for the MLN, all cats with FIP were FCoV-positive, whilst an intermediate group of FCoV-positive non-FIP cats (previously evaluated for inflammatory mediators [28]) was available and utilized to characterize the differing responses to disease versus FCoV infection alone. This organ is also at the gateway between enteric and systemic infection [20], thus taking on additional potential significance.

#### 2.4.1. FCoV Levels in Bone Marrow, Spleen, and Mesenteric Lymph Node of Cats with FIP

**Figure 7:**
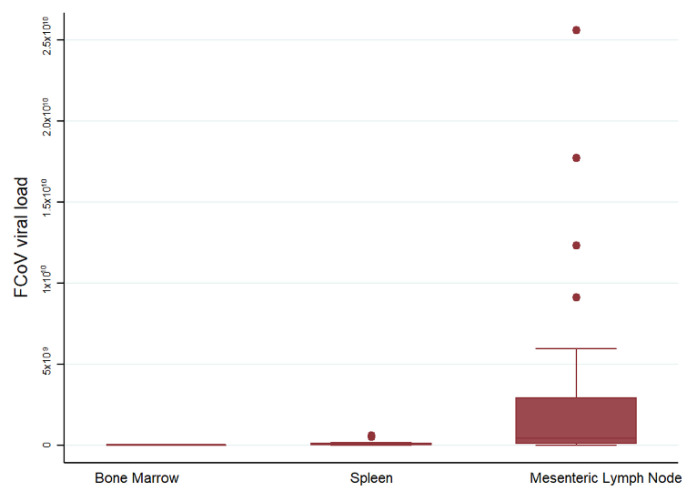
Comparison of relative FCoV mRNA levels between organs. The amount of target was calculated by 2^−ΔΔCT^, using fGAPDH as the internal reference gene for normalization and expressed as an n-fold difference relative to the FIP group mean of the BM samples as a calibrator. The boxes depict the median and interquartile (IQ) range with whiskers extending to the highest and lowest values which are within 1.5× the IQ range. Outliers beyond this are individually marked. There were 11, 14 and 30 samples from each organ respectively.

Mean viral loads were lowest in the BM, and increased in spleen and MLN in cats with FIP (Figure 7). A comparison of viral loads in the MLN of cats with and without FIP confirmed vastly (and significantly) higher loads in cats with FIP. The median 2^ΔΔCt^ was 4.25 × 10^8^ for 30 cats with FIP and 10584.78 for 10 FCoV-positive cats without FIP (*p* < 0.0001).

#### 2.4.2. Changes in G-CSF Transcription Levels in the Bone Marrow, Spleen, and Mesenteric Lymph Node in Cats with FIP

**Figure 8:**
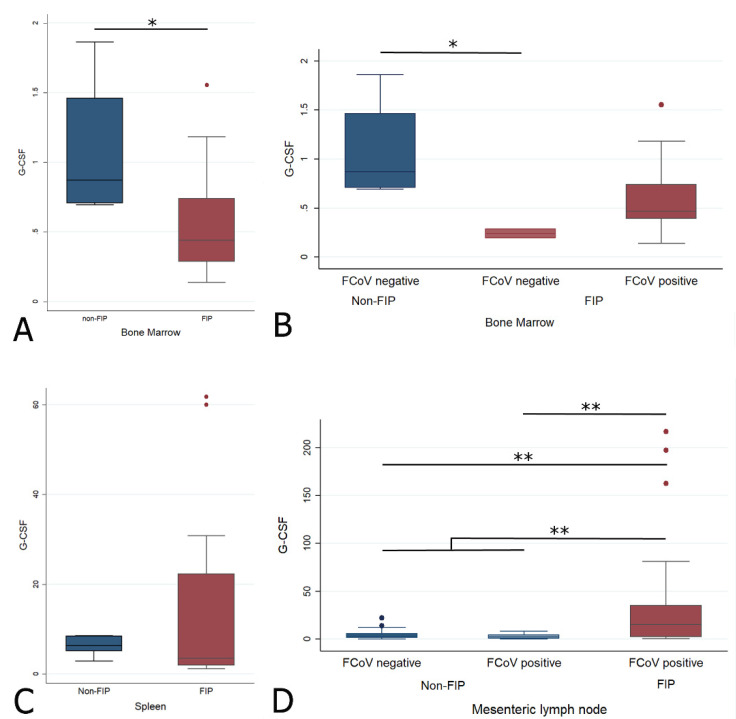
Relative levels of G-CSF transcription across different groups within all organs ((**A**,**B**) bone marrow; (**C**) spleen; (**D**) mesenteric lymph nodes). The amount of target was calculated by 2^−ΔΔCT^, using fGAPDH as the internal reference gene for normalization and expressed as an n-fold difference relative to the non-FIP group mean of the BM samples as a calibrator. The boxes depict the median and interquartile (IQ) range with whiskers extending to the highest and lowest values which are within 1.5× the IQ range. Outliers beyond this are individually marked. */** mark significant differences between groups (*p* < 0.05/*p* < 0.01). FCoV negative: negative qRT-PCR for FCoV; FCoV positive: qRT-PCR for FCoV-positive. FIP: feline infectious peritonitis.

The G-CSF transcription levels were significantly lower in the BM of cats with FIP (Wilcoxon rank sum (Mann–Whitney) (Wrs): z: 2.211, *p* < 0.05, Figure 8A). Interestingly, when FCoV-positive status in the BM was considered, it was the two virus-negative cats with their lower G-CSF levels that mediated the significant difference between disease groups, whereas the difference was not significant when FIP cats with FCoV-positive BM were compared to the non-FIP cats (Figure 8B and Table 1A). There was no significant difference of G-CSF in the spleen (Figure 8C), but cats with FIP had significantly higher levels of G-CSF transcription in the MLN (Figure 8D) (Wrs: z: −3.952, *p* < 0.001). When the different groups of MLN were examined in pairs, both groups without FIP (FCoV-positive and -negative) had significantly lower G-CSF transcription levels than cats with FIP (z: −2.936, *p* < 0.05 and z: −3.534, *p* < 0.001, respectively), whilst there was no significant difference between the non-FIP groups themselves. This showed that there was no significant influence of FCoV infection in the absence of FIP. The results are illustrated in Figure 8 and Table 1.

#### 2.4.3. Changes in M-CSF Transcription Levels in the Bone Marrow, Spleen, and Mesenteric Lymph Node in Cats with FIP

**Figure 9:**
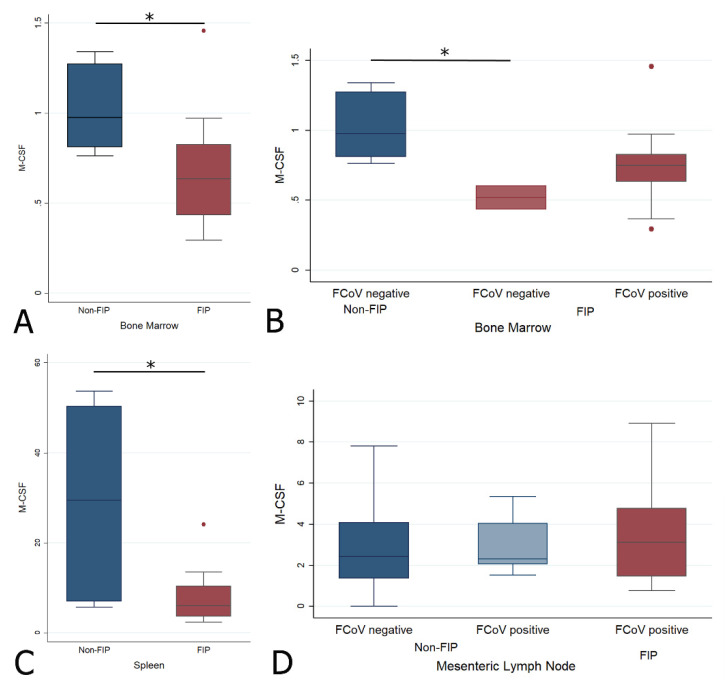
Relative levels of M-CSF transcription across different groups within all organs ((**A**,**B**) bone marrow; (**C**) spleen; (**D**) mesenteric lymph nodes). The amount of target was calculated by 2^−ΔΔCT^, using fGAPDH as the internal reference gene for normalization and expressed as an n-fold difference relative to the non-FIP group mean of the BM samples as a calibrator. The boxes depict the median and interquartile (IQ) range with whiskers extending to the highest and lowest values which are within 1.5× the IQ range. Outliers beyond this are individually marked. */** mark significant differences between groups (*p* < 0.05/*p* < 0.01). FCoV negative: negative qRT-PCR for FCoV; FCoV positive: qRT-PCR for FCoV-positive.

M-CSF was significantly lower in cats with FIP in both the BM (Wrs: z = 2.111, *p* < 0.05) and spleen (Wrs: z = 2.392, *p* < 0.05), with no significant differences between any subgroups for the MLN (Figure 9, Table 2A). When the BM was split by FCoV-positive status, the only significant difference was between FIP and non-FIP FCoV-negative cats (z = 2.000, *p* < 0.05), as for G-CSF.

#### 2.4.4. Changes in GM-CSF Transcription Levels in the Bone Marrow, Spleen, and Mesenteric Lymph Node in Cats with FIP

**Figure 10:**
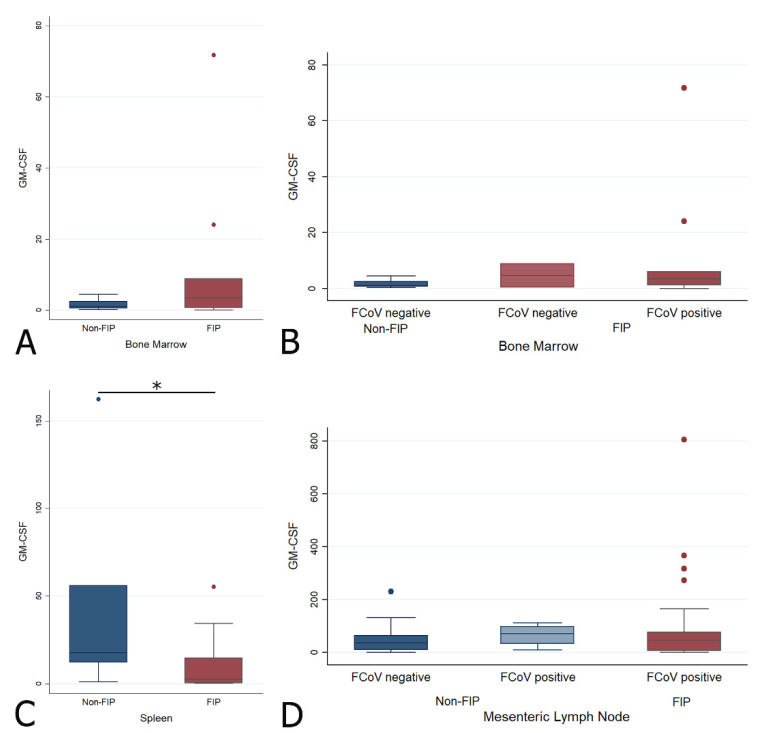
Relative levels of GM-CSF transcription across different groups within all organs ((**A**,**B**) bone marrow; (**C**) spleen; (**D**) mesenteric lymph nodes). The amount of target was calculated by 2^−ΔΔCT^, using fGAPDH as the internal reference gene for normalization and expressed as an n-fold difference relative to the non-FIP group mean of the BM samples as a calibrator. The boxes depict the median and interquartile (IQ) range with whiskers extending to the highest and lowest values which are within 1.5× the IQ range. Outliers beyond this are individually marked. */** mark significant differences between groups (*p* < 0.05/*p* < 0.01). FCoV negative: negative qRT-PCR for FCoV; FCoV positive: qRT-PCR for FCoV-positive.

The spleen showed a significantly lower GM-CSF transcription level in FIP (Wrs: z = 1.979, *p* < 0.05) (Figure 10C). No significant difference was found for the BM or MLN between the main or sub-groups (Figure 10 and Table 3).

#### 2.4.5. Correlations between FCoV Load and CSFs

**Figure 11:**
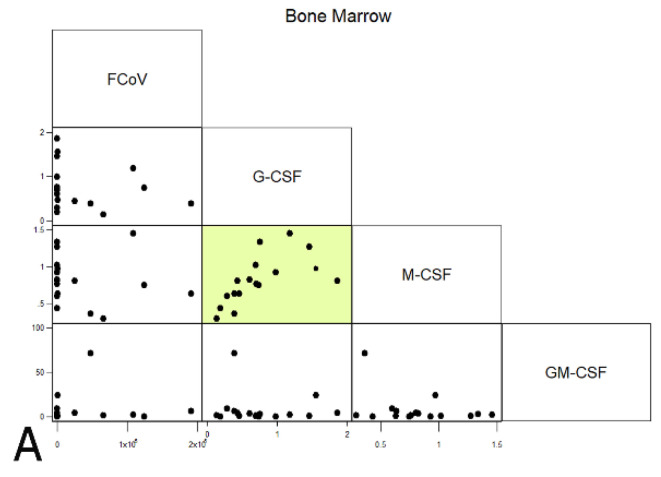

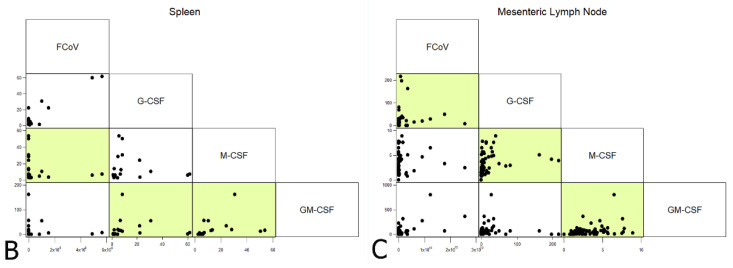
Correlations between FCoV (viral loads) and CSFs (relative mRNA transcription levels) across all cats for each organ ((**A**) bone marrow; (**B**) spleen; (**C**) mesenteric lymph nodes). Highlighted boxes indicate significant correlations.

Levels of CSFs and FCoV were correlated with each other across all cats. Within the BM, G- and M-CSF correlated with each other (rho 0.8064, *p* < 0.0001), which is compatible with both being lower in FIP; no correlations between FCoV and CSF levels were observed. The spleen showed a negative correlation between FCoV and M-CSF (rho −0.4831, *p* < 0.05), as well as positive inter-CSF correlations between GM-CSF and the other CSFs (rho 0.5083, *p* < 0.05 and rho 0.875, *p* < 0.0001, respectively, for G- and M-CSF). In the MLN, the elevated G-CSF transcription in FIP corresponded to a positive correlation with viral load (*p* = 0.0001), whilst M-CSF correlated positively with both other CSFs (rho 0.5014, *p* < 0.0001 and rho 0.3440, *p* < 0.05 for G- and GM-CSF, respectively). The latter correlations were in spite of it being only G-CSF transcription that was significantly upregulated in the MLN in FIP, suggesting that whilst M-CSF and GM-CSF mRNA levels were not altered significantly by the presence of FCoV or by FIP, the stimuli to produce CSFs in the MLN may be linked. The results are presented in Figure 11.

## 3. Discussion

In the present study, we investigated potential changes in relative cytokine transcription levels, focusing on CSFs, to gain information on the direct effect of FIPV on monocytes and macrophages, as well as mediators with direct implications on the function and supply of these cells. We assessed two phases of FIPV infection—early (up to 9 hpi, in vitro) and late, i.e., end-stage FIP (in vivo)—thus avoiding the use of experimental in vivo infection. Immediate post-infection responses were assessed in isolated feline monocytes within the first hours after infection, after the first replication cycles, with FIPV DF2, whilst the late phase response was studied in the hemolymphatic organs of clinical cases euthanized with FIP.

FIPV DF2 is a serotype II coronavirus isolate able to induce FIP experimentally [21,29]. Feline monocytes have been shown to rapidly internalize FCoV via endocytosis and accumulate viral particles in endosomes within 15 min [30], while virus production is seen from 3 to 6 hpi onwards, increasing up to 12 hpi [26]. A previous study has also shown that FIPVs, including the DF2 isolate, rapidly induce p38 MAPK activation in feline peripheral blood mononuclear cells (PBMCs), likely early during entry, with another, lower peak between 6 and 12 hpi [23]. The p38 MAPK pathway is utilized for pro-inflammatory cytokine signaling (IL-1β, TNF-α) [31], and therefore its activation would be expected to correlate with increased levels of these cytokines. For the present study, the time period of 3 to 9 hpi was chosen for two reasons. Firstly, to observe any potential immediate effect of FCoV on the infected cells, and secondly to attempt to minimize (without being able to entirely avoid) any enhancing or suppressing effects by virus particles/proteins and/or cytokines released into the media. The use of monocytes from cats without any previous viral exposure ruled out any potential antibody-dependent enhancement (ADE) effects. FCoV infection was confirmed in all DF2-incubated samples, by RT-qPCR for viral RNA [22].

We observed limited and transient changes in cytokine transcription after infection, i.e., upregulation of IL-6 at 3 hpi, and of GM-CSF at 3 and 6 hpi, and downregulation of M-CSF at the latest time point (9 hpi). The age of the animals at the time of blood collection had a limited effect, showing the more intense IL-1β response of monocytes from younger and older cats, and higher IL-10 mRNA levels in the monocytes of younger cats during the first six hours after infection. These findings reflect the observations made regarding the constitutive transcription of these cytokines [27]. The findings suggest that the FIPV infection of naïve monocytes alone is not sufficient to induce significant activation and thereby the development of FIP lesions. Considering that monocyte-associated viremia is a general consequence of FCoV infection, including in animals that remain healthy [7,8], this was an expected finding. Nonetheless, the observed subtle changes are likely of relevance. We saw significant IL-6 upregulation in monocytes at 3 hpi. For comparison, in PBMCs at 24 hpi, there was no evidence of IL-6 production [23]. These results suggest that as the only potentially pro-inflammatory cytokine upregulated immediately after infection, IL-6 mainly exerts its effect on the monocyte/macrophage pool—IL-6 is known to induce the proliferation of myeloblasts, promyelocytes and colony forming unit-megakaryocyte (CFU-M) [32]. This effect would coincide with that of GM-CSF, which was found to be upregulated at the same time and still at 6 hpi, and is known to induce the proliferation and differentiation of monocyte and neutrophil precursors and to activate mature monocytes and neutrophils, for example by enhancing the expression of some adhesion molecules, such as CD11a and b [33]. The functions of IL-6 also include both increasing the neutrophil pool and increasing their function and lifespan [34]. This may represent an immediate response of the immune system to try and remove the virus. GM-CSF also induces an inflammatory profile in human monocytes; should it have a similar role in cats, this would decrease the required stimulus threshold for activation [35]. In concert, IL-6 and GM-CSF released from newly infected monocytes could increase the monocyte pool and thereby ensure that sufficient cells are available to maintain viral replication. Experimentally, both have biphasic half-lives, with a rapid drop within minutes followed by a more prolonged half-life of around an hour [36,37]; if this situation is replicated in vivo and in felines this could explain why later measurements fail to detect an elevation despite evidence of their downstream effects. Of particular interest regarding the known age susceptibility of young animals to FIP, we found a tendency for GM-CSF transcription levels to decrease with age in response to infection, and a tendency (though neither reached significance) for IL-6 to be transcribed at higher levels after infection in young and old cats vs. middle aged ones. FCoV infection itself is known to not be the key event in disease development; the pro-inflammatory effects of GM-CSF and IL-6 on both monocytes and neutrophils may contribute to susceptibility to lesion development (in which neutrophils contribute to tissue injury despite not being themselves infected). The higher constitutive transcription of IL-1β in younger cats is also in line with their increased propensity to develop FIP [27]; this is further supported by the present findings, i.e., its significantly higher transcription after FIPV infection in the monocytes of young and old cats. We observed significant M-CSF downregulation at the latest time point, 9 hpi. Considering that, apart from its effect on the proliferation of macrophage precursors and the differentiation of monocytes/macrophages, M-CSF also activates macrophages, for example by inducing the expression of other cytokines, such as IL-1β, TNF-α and G-CSF, our results indicate that by downregulating the transcription of M-CSF, the virus would also inhibit the immediate pro-inflammatory activity of the monocytes. Accordingly, we observed age-dependent significantly lower IL-1β and G-CSF transcription after infection, alongside M-CSF downregulation at 9 hpi compared to 3 and 6 hpi, respectively, and no changes in the transcription of any other examined cytokine, i.e., TNF-α, IL-12p40 and IL-10 within the examined time frame. The lack of change in both TNF-α and IL-10 transcription corresponds to the finding in human monocytes that the production of IL-10 is dependent on that of TNF-α, and only begins 8–24 h post experimental addition of TNF-α [38]. These results differ from those of studies on isolated FIPV-infected feline alveolar macrophages or PBMCs. In the latter, TNF-α and IL-1β upregulation was observed at 24 hpi [23], whereas in the former, TNF-α upregulation was seen as a consequence of viral replication at 48 and 72 hpi, and VEGF, G-CSF and GM-CSF were found to be upregulated at 72 hpi; all upregulation levels became significant when virus inoculation was in combination with an antibody against FCoV S protein that is known to induce ADE [18,19]. Interestingly, a study to assess the effect of FCoV infection on the cytokine transcription levels of PBMCs in cats with and without FIP observed increased IL-1β transcription in infected, healthy cats, together with an increase in IFN-γ. This was considered a consequence of CD8+ T cell increase. In contrast, cats with FIP showed reduced IFN-γ transcription, which correlated with the observed drop in CD8+ cells [39]. Taken together, these in vivo and in vitro studies indicate that naïve feline monocytes/macrophages can upregulate pro-inflammatory and monocyte/macrophage colony stimulating cytokines as a consequence of sustained FIPV infection, but that significant upregulation requires further stimulation, such as ADE, for most of these factors apart from TNF-α and IL-1β. IL-1β may, as indicated at least for its presence in natural FCoV infection, be beneficial for the infected cat [39], which provides further support for the relevance of ADE in the development of FIP.

Focusing on the changes occurring in vivo during late-stage disease, there was variation between organs. Both M- and G-CSF were lower in the BM of cats with FIP, but the difference was significant only in FIP cats with FCoV-negative BM samples. This may indicate a systemic attempt at the downregulation of inflammatory mediators that is slightly counteracted locally when the virus is present within the monocyte population of the BM; the BM exhibits an increase in monocytes with systemic FCoV infection and, even more so, with FIP [11]. M-CSF and GM-CSF were lower in the spleen, whilst G-CSF was higher in MLN; the latter is the only CSF that we identified as elevated in FIP. In a previous study, we found slight (but not significant) elevations in IL-6 and TNF-α in the FCoV-infected MLN of cats without FIP, representing a possible intermediate stage. Infected cats have also been shown to exhibit an increase in macrophage numbers in the MLN; therefore, increased CSF levels, contributing to this elevation, were hypothesized [11]. However, there was no difference seen in the FCoV-infected non-FIP group for any CSF, and this may reflect the late timepoint post infection and/or end-stage disease, with a loss of proliferative stimuli that may have been present at an earlier stage.

Within these hemolymphatic organs, in FIP there is an increasing viral load from BM to spleen to MLN. BM alterations reflect most closely the systemic background response, as this organ has not been reported to develop FIP lesions (possibly as the immature monocytes are not susceptible to infection—which instead occurs in circulating and fully differentiated cells) [3]. The spleen may be thought of as intermediate between the two, being highly exposed to circulating effects as well as susceptible to local lesion development, whilst the MLN is commonly a site of typical lesion development [10]. MLN responses may therefore also correlate with a more local and direct response to viral infection. This could be interpreted as exemplifying the host vs. virus battle. Where there is a low level of virus (BM), the host dominates and tries to suppress cytokine production (further supported by lower levels in FCoV-negative samples) and, as regards the CSFs, reduce the supply of host cells to the virus. At intermediate viral levels (as in the spleen), the host is only able to suppress the more homeostatic and less inflammatory M-CSF, whilst at higher levels, as in the MLN, the virus overcomes host opposition to trigger increased G-CSF and help fuel its own source of host cells.

## 4. Materials and Methods

### 4.1. Cases

#### 4.1.1. Blood Donors

This part of the study was performed on 14 barrier-maintained cats, ranging in age from 10 months to 7 years 9 months at the time of sampling. Four cats were male neutered and had been purchased at the age of 1.5 years (Ruprechts Karls University Heidelberg, Zentrales Tierlabor), another four were female and aged 8 months when purchased (Iffa-Credo, L’Arbresle, Cedex, France). The remaining six cats were male neutered and had been purchased at Ciba Geigy, Switzerland, at the age of 4 months.

All cats tested negative for feline leukemia virus, feline immunodeficiency virus, feline herpesvirus, feline calicivirus and parvovirus. Throughout the experiment, cats were housed under barrier-maintained conditions at the Small Animal Clinic, University of Giessen, Germany and at the Animal Facility of the Vetsuisse Faculty, University of Zurich, Switzerland. They were monitored daily for any signs of illness. The animal work was undertaken with permission of the Regierungspräsidium Giessen (II 25.3/19c20/15c) and the Veterinäramt Zürich (118/99).

#### 4.1.2. Clinical Cases

All cats in this part of the study had been seen initially as patients at the university small animal clinics or local veterinary practices of Bristol, UK, or Zurich, Switzerland, and were euthanized with or without FIP for clinical reasons unrelated to this study. A post-mortem examination was performed on each cat with owner consent and samples of BM, spleen and MLN were collected into RNAlater^®^ (Qiagen, Hombrechtikon, Switzerland) within 2 h of death and stored at −80 °C until use. The Bristol cases were part of a biobank, built up as a resource for multiple studies, many of which have been previously utilized [28,40,41]. Animals were grouped according to the presence or absence of FIP, as determined by clinical history, gross examination, histological and immunohistological (demonstration of FCoV antigen within lesional macrophages) findings [42]. Not all tissues were available from all cases: BM was obtained from six non-FIP cats (juvenile to adult) and 11 FIP cats (4 months to 4 years), spleen from six non-FIP (as above) and 14 FIP cats (4 months to 10 years old), and MLN from 40 non-FIP and 30 FIP cases. Based on FCoV RT-qPCR results in the MLN (see below), non-FIP cases were split into FCoV-positive and -negative, resulting in 10 cats with FCoV-positive MLN (these cats did not overlap with those in the spleen or BM groups). Unfortunately, from non-FIP cases with FCoV-positive MLN the other organs were not available, therefore it is not known whether the spleen and BM from these cats were also FCoV-positive.

### 4.2. In Vitro Infection Studies

Blood samples were taken from donors at 4-week intervals. From most animals, two samples were taken at different time points, with two animals being sampled three times. The mean age of the cats whose samples were examined at 3 hpi (n = 25) was 2.64 years (95% CI: 2.00–2.91), at 6 hpi (n = 19) was 2.37 years (95% CI: 1.61–3.14) and at 9 hpi (n = 18) 2.29 years (95% CI: 1.46–3.12).

Each time, 14 mL of peripheral blood was collected and divided into two. From each half, peripheral blood mononuclear cells (PBMCs) were isolated and cultured short-term for 15 to 18 h in Roswell Park Memorial Institute (RPMI) (PAA Laboratories GmbH) supplemented with 10% fetal calf serum (FCS) (PAA Laboratories GmbH) and 1% Pen/Strep (PAA Laboratories GmbH), followed by thorough washing of plates to eliminate non-adherent cells and obtain pure monocytes, as previously described [12,27]. One of the monocyte cultures was then incubated with FIPV DF2 (25 TCID_50;_ [21] for 1 h at 37 °C in 5% CO_2_, followed by washing with RPMI medium without FCS and further incubation in RPMI medium with 10% FCS for another 2 h, 5 h and 8 h, respectively. The other culture received the same treatment with the exception of a sham infection step (uninfected controls). After further washing with medium without FCS, cells were collected in a total of 700 μL RLT buffer (RNeasy Mini Kit, Qiagen, Hilden, Germany) with 1% mercaptoethanol and frozen at −80 °C.

### 4.3. Quantitative Reverse Transcriptase PCR

#### 4.3.1. RNA Extraction

After thawing and addition of 700 μL ethanol, RNA was extracted from monocytes using a commercially available kit (RNeasy Mini kit, Qiagen, Hilden, Germany; [12,27]). The same kit was used for tissue samples, with a prior homogenizing step of the tissue in RLT buffer. Tissue and buffer were placed in a 2 ml round-bottomed tube with a 5 mm steel bead (Retsch, Haan, Germany) and tissues were disrupted using a tissue homogenizer (Mixer-Mill 300, Retsch, Haan, Germany) set to 30 Hz for 40 s before on-column extraction and elution of RNA.

#### 4.3.2. RT-qPCR for FCoV RNA

For the demonstration of FCoV infection, from both monocytes and tissue samples, a TaqMan RT-qPCR detecting the FCoV 7b gene was used as previously described [22,28].

#### 4.3.3. DNAse Treatment, Reverse Transcription and TaqMan qPCR for Feline G-CSF, M-CSF and GM-CSF

Contaminating genomic (g)DNA in RNA extracts was digested and complement (c)DNA synthesized according to published protocols [12]. Real-time TaqMan PCR, using an automated fluorometer (ABI Prism 7700 Sequence Detection System, Applied Biosystems, Weiterstadt, Germany), allowed for the relative quantification of feline (f)IL-1β, fIL-6, fIL-10, fIL-12p40, fTNF-α, fG-CSF, fM-CSF and fGM-CSF transcription [12,22,27]. From every cDNA sample, parallel reactions were performed in duplicate. Feline housekeeping gene glyceraldehyde 3-phosphate dehydrogenase (fGAPDH) served as an internal control for normalization. Amplification conditions, assay compositions, primer, and probe concentrations were as previously described [12,27].

### 4.4. Quantification of Cytokine Transcripts

The relative quantification of cytokine signals was done by the comparative Ct method [43]. The Ct value represents the PCR cycle at which an increase in reporter fluorescence exceeds 10× the standard deviation of the first detectable baseline fluorescence. For each sample, differences between the target and internal control Ct were calculated and served to normalize differences in the amount of total nucleic acid added to each reaction and the efficiency of the RT step. The Ct of the internal control, fGAPDH, was subtracted from the Ct of each experimental sample: ΔCt value. For samples that did not yield a cytokine signal after 45 cycles, a ΔCt was created by subtraction of the lowest Ct for fGAPDH (i.e., obtained from the sample with the highest transcription of GAPDH) from 45 (total number of cycles run). The resulting value was rounded to the next highest integer (20).

Differences in cytokine transcription between uninfected monocytes and infected monocytes for the different time points were also evaluated as follows: For each cytokine, the Ct value obtained from uninfected monocytes was subtracted from the ΔCt values obtained from infected monocytes (ΔΔCt value). Cytokine transcription was calculated as the difference in transcription of the respective cytokine from control monocytes and experimental monocytes: 2^−ΔΔCt^ [12,27,43].

Tissue sample results were calculated slightly differently in that there was no paired control sample. Here, the mean bone marrow ΔCt from the non-FIP group was used as the calibrator to obtain ΔΔCt, following the same initial step of comparing the target and GAPDH Ct values. Analysis and graphical representation were performed on 2^−ΔΔCt^.

### 4.5. Statistical Analysis

All statistical analyses were completed using Stata 13.1 (StataCorp LP). For the in vivo study, non-parametric methods (Mann–Whitney/Wilcoxon rank test and Spearman’s correlation) were used to examine differences in viral load and cytokine expression between FIP and non-FIP specimens. For the in vitro study, initial analysis of the ΔCt values used univariate analysis (*t*-test and ANOVA) to examine preliminary associations with time of infection and differences between infected and uninfected cells. Further analysis of the associations between age and infection time with the cytokine transcription used log10 transformation of the ΔΔCt values of the difference between control and infected cell cultures, which led to approximate normal distributions. Log-transformed values above zero indicated increased cytokine expression in the infected cells with negative values indicating a decrease. Cytokine expression was examined against time from the point of infection and the age of the animals using univariate analysis (t-test). To examine and visualize the association between age and upregulation of cytokine transcription, paired animal samples were organized in six age groups of approximately equal number (group 0 (n = 10): 10 to 13 months old; group 1 (n = 7): 14 to 15 months old; group 2 (n = 14): 16 to 21 months old; group 3 (n = 10): 22 to 28 months old; group 4 (n = 10): 29 to 36 months old and group 5 (n = 11): 59 to 93 months old). Both linear and quadratic multivariate regression models were examined using cluster (animal id) robust standard errors. Model fitness was assessed by examining the distribution of residual values. The threshold for statistical significance was established at *p* < 0.05.

## 5. Conclusions

In conclusion, our data suggest that FIPV infection of feline monocytes has limited immediate effects on cytokine expression by these cells. A potential increase in the monocyte/macrophage pool and limited, short-term neutrophil activation appear to be the most likely main consequences. This may however be associated with increased phagocytosis, and hence contribute to an increased cell uptake and increased viral load even in the absence of notably increased cell numbers [44]. These results suggest that the monocyte-associated viremia that develops in the vast majority of infected cats may elicit similar effects, supported by the limited increase in the monocyte/macrophage populations observed in healthy, FCoV-infected cats [6,11,45]. For the significant upregulation of other, including pro-inflammatory, cytokines, it is likely that more substantial viral replication, as is seen in cats with FIP [11] and in vitro FCoV monocyte infection studies [26], further stimulation, e.g., through ADE [18,19,29,46], cell–cell interactions or other so far unknown factors (such as viral mutations), are required. These are then also associated with a more substantial increase in the macrophage population [10]. In end-stage FIP, CSF levels in hemolymphatic organs are higher in those organs with a higher viral load, suggesting their elevation is a direct response.

## Figures and Tables

**Table 1:** A and B: Significance levels of all Wilcoxon rank sum pairwise comparisons between groups and subgroups (**A**), with group medians (**B**) for G-CSF.

**A**	**Organ**	**Comparison**	**Probability**
	BM	FIP vs. non-FIP	<0.05 *
		FIP FCoV +ve vs. FIP FCoV −ve	0.099
		FIP FCoV +ve vs. non-FIP	0.059
		FIP FCoV −ve vs. non-FIP	<0.05 *
	Spleen	FIP vs. non-FIP	0.564
	MLN	FIP vs. non-FIP	<0.01 *
		FIP vs. non-FIP FCoV +ve	<0.01 *
		FIP vs. non-FIP FCoV −ve	<0.01 *
		non-FIP FCoV +ve vs. non FIP FCoV −ve	0.574
**B**	**Organ**	**FIP Median (n)**	**Non-FIP Median (n)**
	BM	25,158.38 (11)	0.872 (6)
	Spleen	1.03 × 10^7^ (14)	6.298 (6)
	MLN	4.25 × 10^8^ (30)	all cases 3.082 (40)
			FCoV +ve 2.249 (10)
			FCoV −ve 3.120 (30)

BM: bone marrow; FIP: feline infectious peritonitis; FCoV: feline coronavirus; MLN: mesenteric lymph node. * indicates a *p* value of at least <0.05.

**Table 2:** A and B: Significance levels of all Wilcoxon rank sum pairwise comparisons between groups and subgroups (**A**), with group medians (**B**) for M-CSF.

**A**	**Organ**	**Comparison**	**Probability**
	BM	FIP vs. non-FIP	<0.05 *
		FIP FCoV +ve vs. FIP FCoV −ve	0.239
		FIP FCoV +ve vs. non-FIP	0.077
		FIP FCoV −ve vs. non-FIP	<0.05 *
	Spleen	FIP vs. non-FIP	<0.05 *
	MLN	FIP vs. non-FIP	0.476
		FIP vs. non-FIP FCoV +ve	0.975
		FIP vs. non-FIP FCoV −ve	0.383
		non-FIP FCoV +ve vs. non FIP FCoV −ve	0.731
**B**	**Organ**	**FIP Median (n)**	**non-FIP Median (n)**
	BM	0.635 (11)	0.975 (6)
	Spleen	5.992 (14)	29.449 (6)
	MLN	3.133 (30)	all cases 2.422 (40)
			FCoV +ve 2.249 (10)
			FCoV −ve 3.120 (30)

* indicates a *p* value of at least <0.05.

**Table 3:** A and B: Significance levels of all Wilcoxon rank sum pairwise comparisons between groups and subgroups (**A**), with group medians (**B**) for GM-CSF.

**A**	**Organ**	**Comparison**	**Probability**
	BM	FIP vs. non-FIP	0.191
		FIP FCoV +ve vs. FIP FcoV −ve	0.814
		FIP FcoV +ve vs. non-FIP	0.157
		FIP FcoV −ve vs. non-FIP	0.739
	Spleen	FIP vs. non-FIP	<0.05 *
	MLN	FIP vs. non-FIP	0.794
		FIP vs. non-FIP FcoV +ve	0.349
		FIP vs. non-FIP FcoV −ve	0.442
		non-FIP FcoV +ve vs. non FIP FcoV −ve	0.075
**B**	**Organ**	**FIP Median (n)**	**Non-FIP Median (n)**
	BM	3.462 (11)	0.965 (6)
	Spleen	2.400 (14)	17.787 (6)
	MLN	46.143 (30)	all cases 41.967 (40)
			FcoV +ve 71.609 (10)
			FcoV −ve 36.472 (30)

* indicates a *p* value of at least <0.05.

## Supplementary Materials

The following are available online at https://www.mdpi.com/2076-0817/9/11/893/s1, Figure S1: Box plots presenting the relative transcription (ΔΔCt log10) of all the cytokines examined in the in vitro study grouped by age and time post infection. The reference line indicates the point where there is no difference between infected and uninfected cells. (Group 0 (n = 10): 10 to 13 months old; group 1 (n = 7): 14 to 15 months old; group 2 (n = 14): 16 to 21 months old; group 3 (n = 10): 22 to 28 months old; group 4 (n = 10): 29 to 36 months old and group 5 (n = 11): 59 to 93 months old), Table S1: Results of ANOVA statistical analyses performed on FIPV-infected and mock (non-)infected monocytes at 3, 6, and 9 hpi for all studied cytokines.
