# Supplementary material for: Colony Stimulating Factors in Early Feline Infectious Peritonitis Virus Infection of Monocytes and in End Stage Feline Infectious Peritonitis; A Combined In Vivo and In Vitro Approach
Source: Pathogens. 2020 Oct 27;9(11):893. doi: 10.3390/pathogens9110893 (PMC7692899; doi:10.3390/pathogens9110893)
Supplement: Supplementary file 1 [file pathogens-09-00893-s001.zip › Supplementary Table 1.docx]

Table S1: Results of ANOVA statistical analyses performed on FIPV infected and mock (non-)infected monocytes at 3, 6, and 9 hpi for all studied cytokines

|  |  | CoV | ΔCt | | ΔCt 3 hpi | | ΔCt 6 hpi | | ΔCt 9 hpi | | ANOVA association with time post infection |
| --- | --- | --- | --- | --- | --- | --- | --- | --- | --- | --- | --- |
|  |  |  | Mean | 95%CI | Mean | 95%CI | Mean | 95%CI | Mean | 95%CI |  |
| IL-1β | Infected | 4.076 | -0.954 | -1.467 - -0.441 | -1.126 | -1.719 - -0.533 | -1.423 | -2.393 - -0.453 | -0.221 | -1.495 - 1.053 | R^2^=0.0587, F=1.84, p=0.1768 |
|  | Non-infected | 6.665 | -1.39 | -2.045 - -0.734 | -0.772 | -1.754 - 0.331 | -1.697 | -2.796 - -0.597 | -2.007 | -3.432 - -0.582 | R^2^=0.0496, F=1.54, p=0.2232 |
| IL-6 | Infected | 24.781 | 5.513 | 4.249 - 6.778 | 4.322 | 3.369 - 5.274 | 4.677 | 2.545 - 6.808 | 8.052 | 4.501 - 11.604 | R^2^=0.109, F=3.61, p<0.05 |
|  | Non-infected | 29.512 | 6.617 | 5.237 - 7.997 | 6.944 | 4.815 - 9.074 | 6.574 | 3.814 - 9.334 | 6.208 | 3.340 - 9.076 | R^2^=0.032, F=0.09, p=0.9102 |
| IL-10 | Infected | 39.413 | 6.101 | 4.507 - 7.695 | 6.962 | 4.370 - 9.554 | 3.623 | 2.102 - 5.144 | 7.521 | 3.509 - 11.534 | R^2^=0.0714, F=2.27, p=0.1126 |
|  | Non-infected | 27.317 | 5.745 | 4.418 - 7.072 | 6.573 | 4.103 - 9.043 | 5.724 | 3.814 - 9.334 | 4.617 | 2.157 - 7.077 | R^2^=0.024, F=0.73, p=0.488 |
| IL-12 p40 | Infected | 35.584 | 11.646 | 10.131 - 13.161 | 10.941 | 8.633 - 13.249 | 11.511 | 8.764 - 14.257 | 12.769 | 9.357 - 16.181 | R^2^=0.0163, F=0.49, p=0.6150 |
|  | Non-infected | 30.702 | 10.453 | 9.046 - 11.860 | 9.511 | 7.749 - 11.273 | 11.680 | 8.696 - 14.664 | 10.467 | 7.289 - 13.646 | R^2^=0.0271, F=0.82, p=0.4443 |
| TNF-α | Infected | 17.59 | 3.836 | 2.771 - 4.901 | 3.018 | 2.245 - 3.792 | 4.237 | 2.013 - 6.461 | 4.549 | 1.653 - 7.445 | R^2^=0.0270, F=0.82, p=0.4466 |
|  | Non-infected | 17.58 | 3.82 | 2.756 - 4.885 | 3.506 | 2.549 - 4.463 | 3.444 | 1.220 - 5.609 | 4.654 | 1.793 - 7.515 | R^2^=0.0165, F=0.49, p=0.6126 |
| G-CSF | Infected | 48.646 | 3.89 | 2.119 - 5.662 | 4.560 | 1.634 - 7.485 | 0.564 | -0.580 - 1.708 | 6.471 | 2.091 - 10.852 | R^2^=0.1150, F=3.83, p<0.05 |
|  | Non-infected | 38.761 | 4.355 | 2.774 - 5.937 | 5.462 | 3.077 - 7.848 | 2.008 | 0.429 - 3.588 | 5.296 | 1.086 - 9.506 | R^2^=0.064, F=2.02, p=0.1423 |
| M-CSF | Infected | 27.051 | 5.349 | 4.029 - 6.670 | 2.859 | 2.224 - 3.493 | 6.762 | 3.255 - 10.269 | 7.317 | 4.973 - 9.661 | R^2^=0.1592, F=5.59, p<0.01 |
|  | Non-infected | 29.081 | 4.528 | 3.159 - 5.898 | 2.862 | 2.123 - 3.602 | 7.886 | 4.247 - 11.525 | 3.299 | 1.009 - 5.588 | R^2^=0.1752, F=6.27, p<0.01 |
| GM-CSF | Infected | 27.356 | 5.609 | 4.281 - 6.938 | 4.778 | 3.810 - 5.746 | 3.223 | 2.013 - 4.432 | 9.284 | 5.366 - 13.201 | R^2^=0.2208, F=8.36, p<0.001 |
|  | Non-infected | 36.272 | 8.401 | 6.878 - 9.937 | 8.861 | 6.527 - 11.195 | 7.176 | 4.592 - 9.760 | 9.075 | 5.485 - 12.665 | R^2^=0.019, F=0.57, p=0.5685 |
