# Supplementary figures and images for: Colony Stimulating Factors in Early Feline Infectious Peritonitis Virus Infection of Monocytes and in End Stage Feline Infectious Peritonitis; A Combined In Vivo and In Vitro Approach
Source: Pathogens. 2020 Oct 27;9(11):893. doi: 10.3390/pathogens9110893 (PMC7692899; doi:10.3390/pathogens9110893)

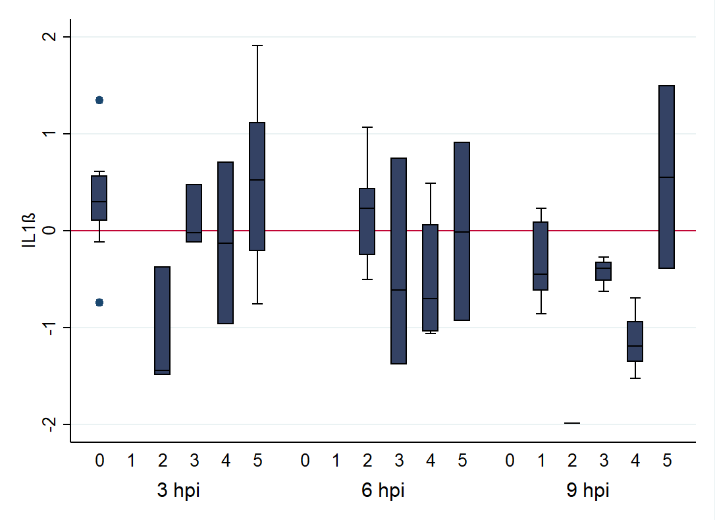

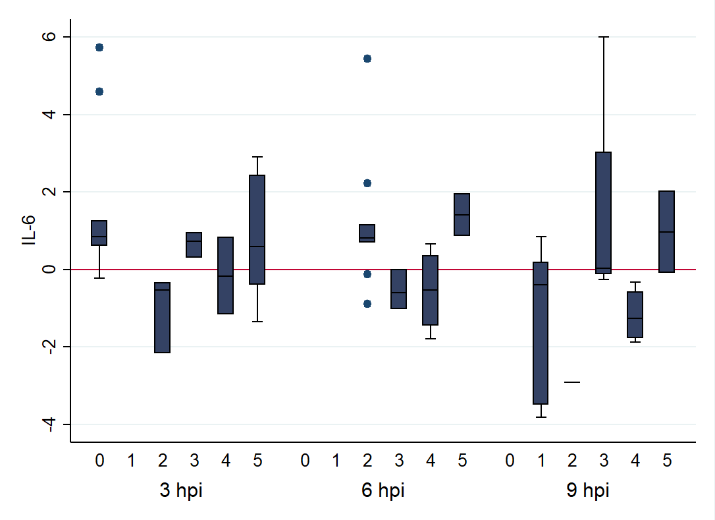

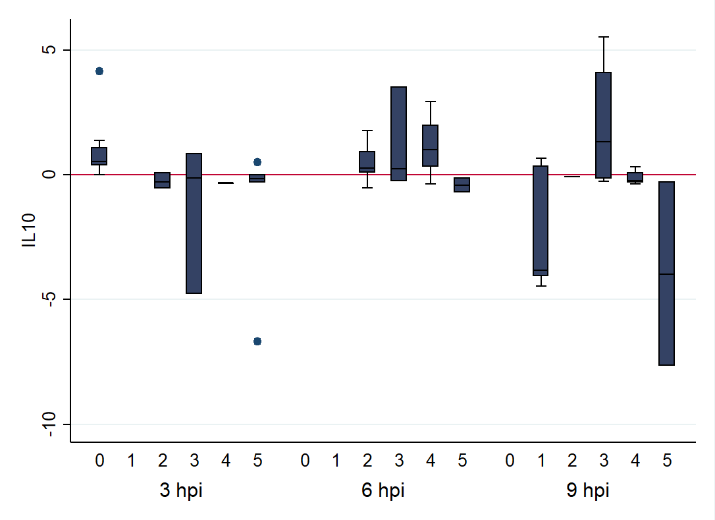

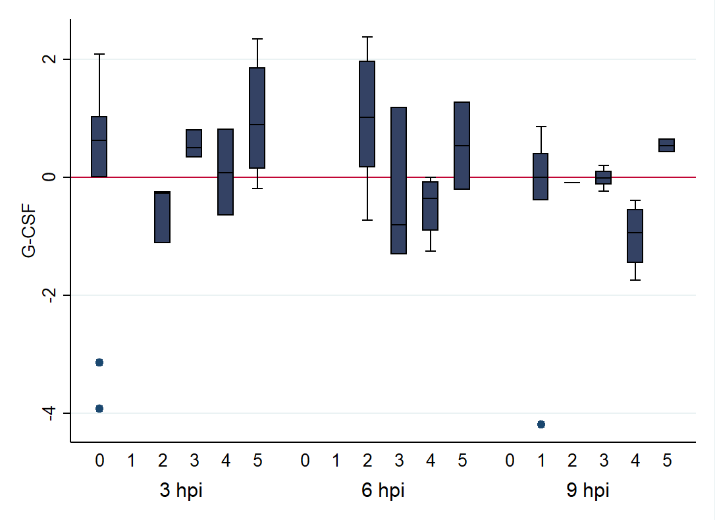

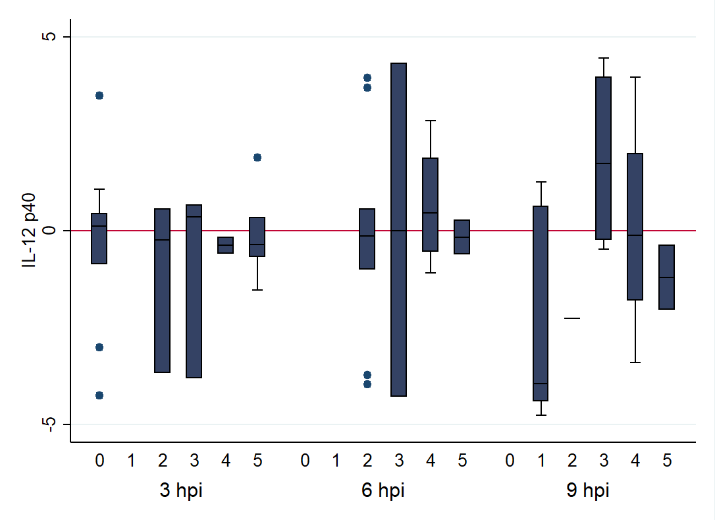

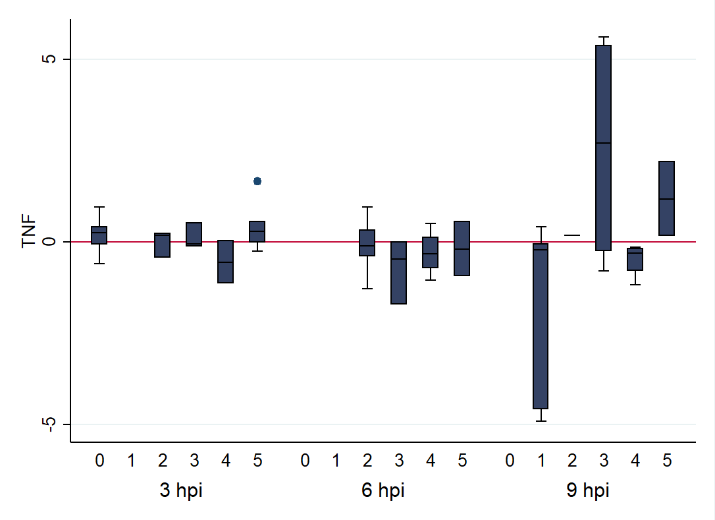

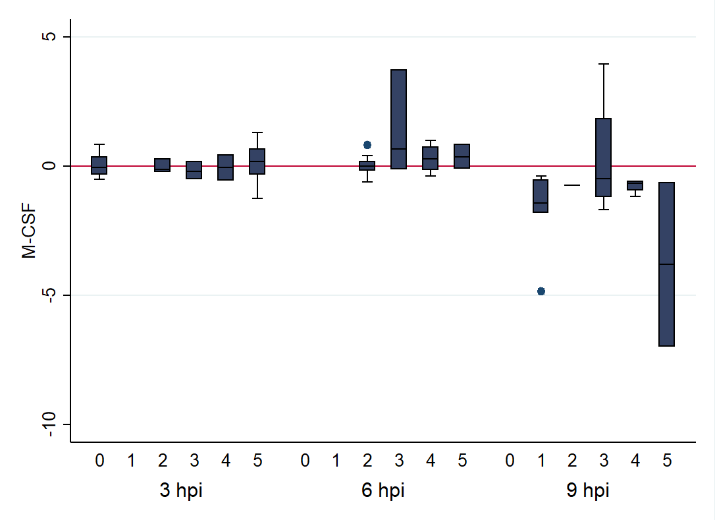

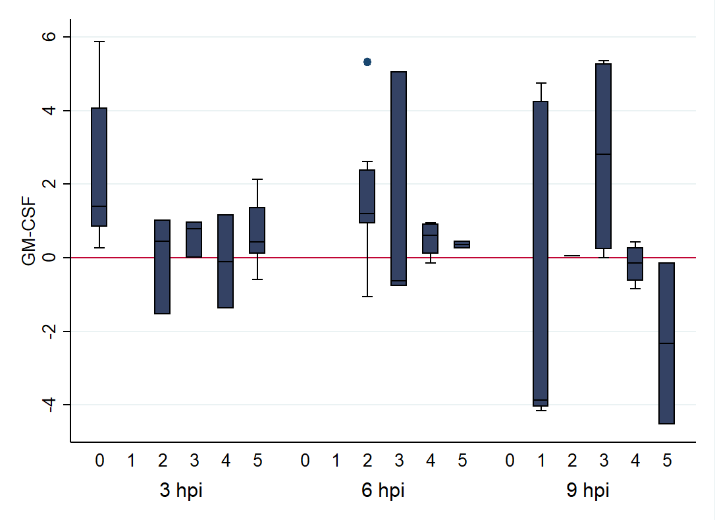

Supplement: Supplementary file 1 [file pathogens-09-00893-s001.zip › Supplementary Figure 1.docx]
